# Ubiquitination of Metabotropic Glutamate Receptors and Associated Synaptic Proteins *In Vitro* and *In Vivo*

**DOI:** 10.33696/Signaling.6.145

**Published:** 2025

**Authors:** Li-Min Mao, John Q. Wang

**Affiliations:** 1 Department of Biomedical Sciences, University of Missouri-Kansas City, School of Medicine, Kansas City, MO, USA; 2 Department of Anesthesiology, University of Missouri-Kansas City, School of Medicine, Kansas City, MO, USA

**Keywords:** mGlu, Glutamate, Homer, PICK1, Ubiquitin, Ubiquitination, Proteasome, Degradation

## Abstract

Metabotropic glutamate (mGlu) receptors are a family of G protein-coupled receptors. These receptors are widely distributed in the brain and are critical for the modulation of synaptic transmission and plasticity. Emerging evidence shows that mGlu receptors themselves are subject to a dynamic posttranslational modification involving protein ubiquitination. Postsynaptic group I mGlu receptors (mGlu1/5) undergo constitutive ubiquitination at lysine sites on their intracellular domains in heterologous cells and neurons. In particular, ligand stimulation triggers rapid ubiquitination of group I receptors to confer a negative feedback regulation. Like mGlu1/5, presynaptic mGlu7 receptors are ubiquitinated by a specific E3 ubiquitin ligase. Robust ubiquitination is also seen in a number of existing synaptic proteins closely associated with mGlu activity, including Homer1a, the activity-regulated cytoskeleton-associated protein Arc/Arg3.1, and the protein interacting with C-kinase 1. Ubiquitination of mGlu receptors targets the receptors to degradation via the proteasome-dependent or -independent pathway or plays nondegradative roles in the regulation of distinct cellular processes such as endocytic trafficking, protein-protein interactions, and mGlu receptor signaling.

## Introduction

The neurotransmitter L-glutamate interacts with ionotropic and metabotropic glutamate (mGlu) receptors in the mammalian brain [1]. mGlu receptors are a family of class C G protein-coupled receptors, and eight mGlu receptor subtypes are subdivided into three functional groups (I-III). Group I receptors (mGlu1/5) are coupled to heterotrimeric G_q_ proteins. Activation of them causes phospholipase Cβ1 to hydrolyze phosphoinositide into inositol 1,4,5-trisphosphate and diacylglycerol, leading to Ca^2+^ release and protein kinase C (PKC) activation [2,3]. Group II (mGlu2/3) and group III (mGlu4/6/7/8) receptors are coupled to G_i/o_ proteins. Their activation leads to inhibition of adenylyl cyclase and thus reduction of cAMP formation and protein kinase A activity. In addition to these canonical signaling pathways, mGlu receptors modulate many other signaling molecules and ion channels. Group I receptors are primarily localized at postsynaptic sites, while group II/III receptors are principally presynaptic. As a set of receptors that are widely distributed throughout the brain, mGlu receptors are actively involved in the regulation of numerous neuronal and synaptic activities and are linked to various neurological and neuropsychiatric disorders [2,3].

**Figure 1.**
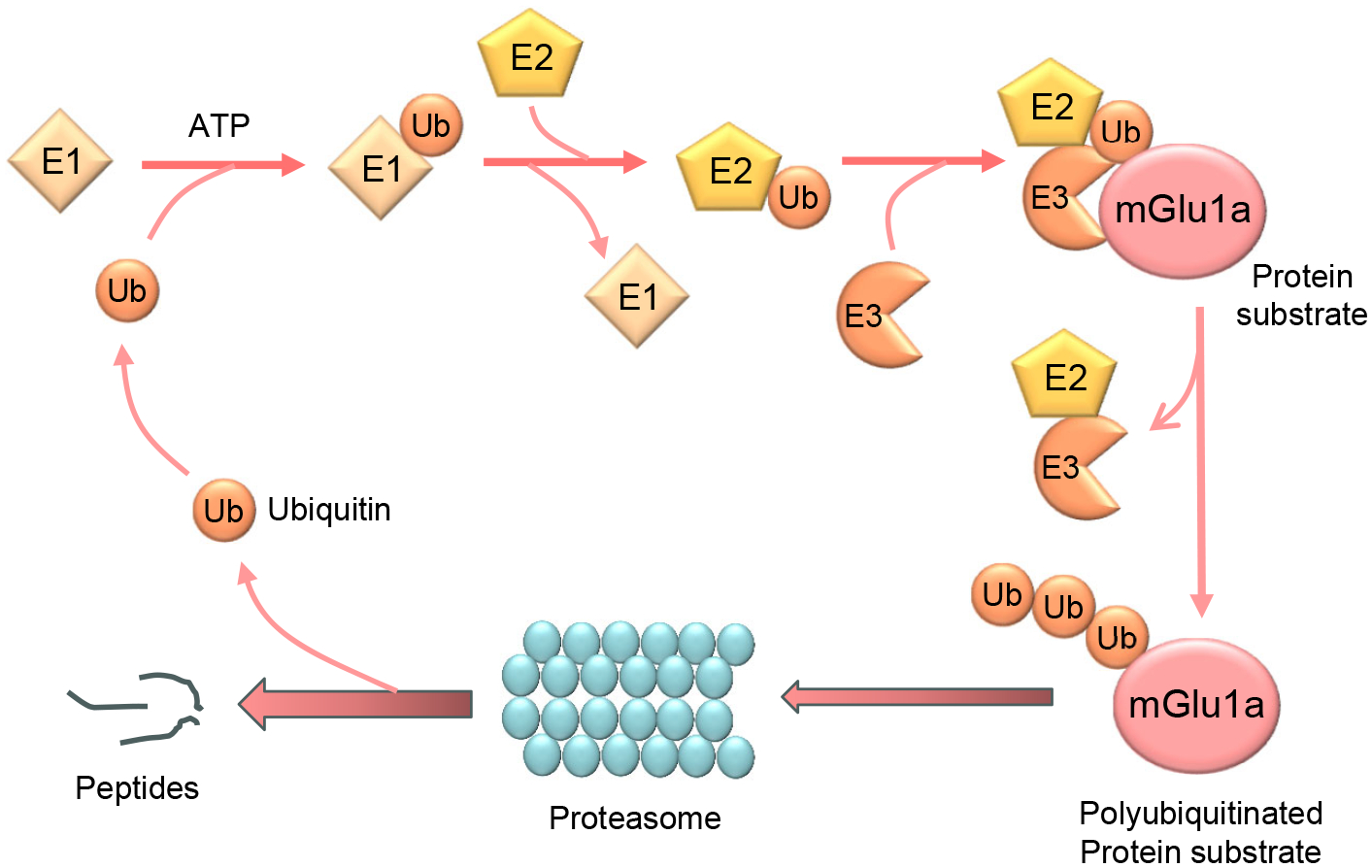
A schematic diagram illustrating a three-step enzymatic cascade for protein ubiquitination. The cascade starts with activation of ubiquitin (Ub) by a ubiquitin-activating enzyme (E1). In this step, ATP is involved. The ubiquitin is then transferred from the ubiquitin-E1 complex to a ubiquitin-conjugating enzyme (E2). Subsequently, the ubiquitin in the ubiquitin-E2 conjugate is transferred to a lysine residue in a substrate such as mGlu1a directly from the E2 with a mediating role played by a RING-finger-containing E3 ligase. This process repeats until the target protein has a ubiquitin chain. A polyubiquitinated substrate is then directed to the proteasome for degradation.

Ubiquitination is an important posttranslational modification for regulating function of existing proteins under constitutive and activity-dependent conditions [4,5]. As a tightly regulated enzymatic cascade (Figure 1), protein ubiquitination starts with activation of ubiquitin, a highly conserved 76-amino acid polypeptide, by the ubiquitin-activating enzyme (E1). Active ubiquitin is then transferred to the ubiquitin-conjugating enzyme (E2). Through the ubiquitin ligase (E3), the last C-terminal amino acid of ubiquitin (G76) binds to mostly a lysine residue on the target protein via a covalent isopeptide bond. In this step, one of the hundreds of E3 ligases confers specificity to the ubiquitination reaction. Typically, ubiquitination repeats itself until the assembly of a polyubiquitin chain on the substrate protein. A ubiquitin K48-linked polyubiquitin chain is the most abundant type of polyubiquitination and is the canonical signal for directing proteins to the 26S proteasome for degradation, while K63-linked polyubiquitination can serve as a reversible posttranslational modification to play nondegradative roles in cellular processes such as inflammatory signaling, DNA repair, endocytosis, etc. or can target proteins to degradation via the autophagy-lysosomal pathway [4,5]. In addition, conjugation of a single ubiquitin molecule to one protein at a single residue (i.e., monoubiquitination) or multiple residues (multi-monoubiquitination) can affect the trafficking, interaction, and other activities of tagged proteins in a proteasome-independent manner [6].

Protein ubiquitination has emerged as a robust mechanism for the dynamic regulation of synaptic transmission and plasticity. It is known now that the active ubiquitin-proteasome system (UPS) resides in the presynaptic active zone as well as the postsynaptic density (PSD) and regulates turnover and function of a set of proteins at both pre- and postsynaptic sites [7–10]. mGlu receptors are among a large number of synaptic proteins subjected to ubiquitination. Among the mGlu receptor subtypes investigated so far, mGlu1/5 and mGlu7 have been found to be vigorously regulated by the ubiquitination mechanism. Available evidence shows that constitutive and activity-dependent ubiquitination of presynaptic mGlu7 and postsynaptic mGlu1/5 receptors leads to UPS-dependent or -independent degradation of the receptors or directly affects their endocytosis and function. Functional ubiquitination also occurs to synaptic proteins that are closely associated with mGlu receptors. Taken together, ubiquitination plays degradative or nondegradative roles in controlling stability, trafficking, subcellular distributions, protein-protein interactions, and signaling of mGlu receptors and thus fine-tunes the strength and efficacy of glutamatergic transmission.

## Group I mGlu Receptors

**Figure 2.**
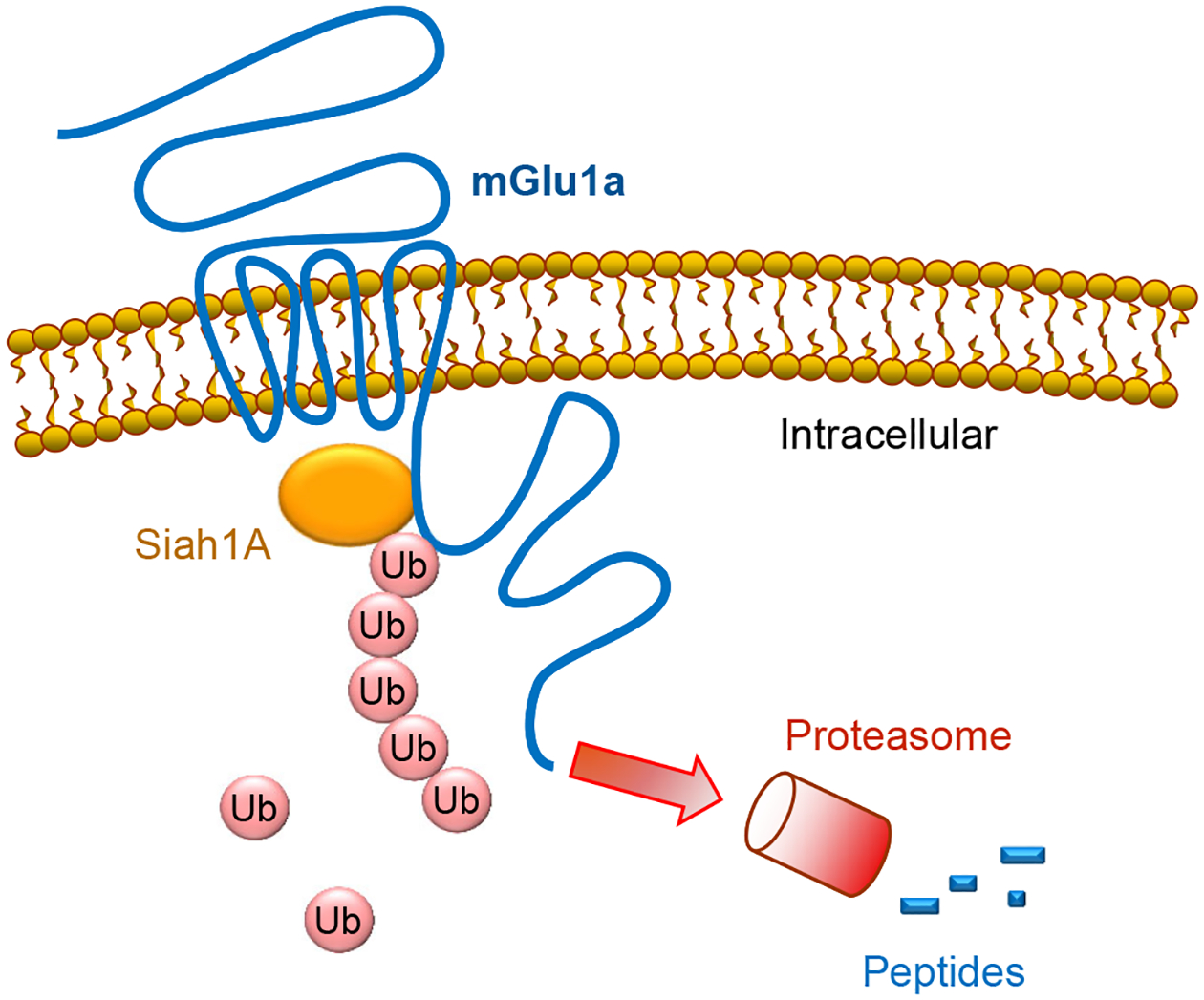
Ubiquitination and degradation of mGlu1a receptors. The E3 ubiquitin ligase Siah1A binds to the proximal region of mGlu1a C-terminus. The Siah1A binding enables Siah1A to serve as a specific E3 ligase to catalyze the binding of ubiquitin (Ub) to existing mGlu1a receptor proteins likely at multiple lysine sites. The Siah1A-induced polyubiquitination of mGlu1a receptors leads to subsequent degradation of the receptors via the 26S proteasome, resulting in acceleration of the mGlu1a protein turnover.

Group I receptors are the first subgroup of mGlu receptors shown to be subject to the regulation by the ubiquitination-dependent mechanism. It is well known that a variety of E3 ligases carry out ubiquitination. Each ligase recognizes a specific target protein and brings it to a discrete fate of either degradation or other cellular processes. In the case of group I receptors, an early study with yeast two-hybrid screening revealed that seven in absentia homolog 1A (Siah1A), a member of the RING-finger-containing E3 ubiquitin ligases, selectively interacted with mGlu1/5 [11]. Siah1A directly bound to the proximal region of mGlu1a/5a C-terminus (CT) (Figure 2), while Siah1A did not interact with mGlu2 and mGlu7 in their CT regions [11]. The Siah1A-interacting sequence is present in the splice variants with long (mGlu1a and mGlu5a/5b) but not short (mGlu1b/1f/1d and mGlu5d) CT domains. The interaction of full-length mGlu1a with Siah1A was confirmed in transfected COS-7 mammalian cells [11] and was activity-dependently induced in hippocampal neurons [12]. Moreover, Siah1A and group I mGlu mRNAs are co-expressed in the same cell populations in the mouse hippocampus and cerebellum.

The interaction between Siah1A and mGlu1/5 may enable Siah1A to serve as a functional E3 ligase to specifically ubiquitinate and degrade existing receptor proteins at the posttranslational level. Indeed, co-transfection of Siah1A induced polyubiquitination of recombinant mGlu1a and mGlu5 proteins in HEK293 cells [13]. Multiple lysine residues spanning from intracellular loops to the CT region of mGlu5 were ubiquitinated by Siah1A (Table 1). The Siah1A-induced polyubiquitination of mGlu1a was causally linked to subsequent degradation of the receptors via the 26S proteasome, resulting in acceleration of the mGlu1a protein turnover and loss of mGlu1a at the protein but not mRNA level in transfected cells [13]. Meanwhile, Siah1A did not alter mGlu3 and mGlu7 expression. Of note, in a heterologous expression cell line (rat sympathetic superior cervical ganglion neurons), co-expressed Siah1A attenuated group I mGlu-mediated inhibition of Ca^2+^ currents, although this effect was likely due to a direct association of Siah1A with group I receptors rather than a result of targeting the receptors to the proteasome [14].

Ubiquitination of group I receptors could be a regulated event. The cystic fibrosis transmembrane conductance regulator-associated ligand (CAL) through its PSD-95/discs large/ZO-1 (PDZ) homolog domain bound to the distal end of mGlu5a CT [15]. As such, CAL inhibited ubiquitination and degradation of mGlu5a and elevated receptor expression at the posttranslational (i.e., protein but not mRNA) level. Unlike CAL, Homer3 positively regulates the proteasomal degradation of mGlu1a receptors. Homer3, a long isoform of the Homer scaffold protein family, but not Homer1/2 bound to S8 ATPase, a subunit of the 26S proteasome [16]. Since Homer3 is known to bind to mGlu1a, Homer3 links mGlu1a to S8 ATPase to form an mGlu1a/Homer3/S8 ATPase complex in neurons *in vivo*. As a result, Homer3 enhances ubiquitination of mGlu1a receptors and delivers the ubiquitinated mGlu1a to the proteasome for degradation.

Ligand activation of group I receptors can trigger a negative feedback loop involving a ubiquitination link. The group I mGlu agonist DHPG induced rapid polyubiquitination of mGlu1 at a K1112 site in HEK293 cells and cultured hippocampal neurons [17]. UBEI-41 (also known as PYR-41), an inhibitor of the E1 activating enzyme in the ubiquitin cascade, and siRNA knockdown of Siah1A blocked the DHPG-stimulated polyubiquitination of mGlu1 receptors. Both UBEI-41 and Siah1a siRNAs also blocked the DHPG-induced endocytosis of mGlu1 receptors, indicating a role of ubiquitination in the agonist-induced internalization of the receptors. In other studies, ligand activation of mGlu5 receptors triggered PKC to phosphorylate a residue (S901) on the mGlu5 CT, which in turn disrupted the binding of calmodulin to mGlu5 and therefore promoted the binding of the E3 ligase Siah1A to the receptors [12,18]. Siah1A then induced monoubiquitination of mGlu5 and sorted the receptors into the late endosomal/lysosomal pathway. The accelerated lysosomal degradation of mGlu5 led to a decrease in expression of mGlu5 receptors in heterologous HeLa cells and cultured hippocampal neurons [12]. The above results generally establish a ubiquitination-dependent feedback model in which agonist stimulation induces ubiquitination of group I receptors, which results in internalization or degradation of the ubiquitinated receptors. Based on this model, inhibition of the negative feedback ubiquitination is reasoned to enhance group I receptor functions. In fact, inhibition of ubiquitination by UBEI-41 or knockdown of Siah1A enhanced a series of DHPG-induced events, including 1) membrane depolarization, 2) phosphorylation of extracellular signal-regulated kinases (ERK), 3) endocytosis of AMPA receptors, and 4) long-term depression (LTD), in heterologous cells or hippocampal neurons [17,19]. Of note, the proteasome inhibitor lactacystin produced the similar effect in these events [19]. Thus, the proteasomal pathway, in addition to the lysosomal pathway, participates in the degradation of group I receptors implicated in the negative feedback regulation of the receptors.

The dynamic balance between ubiquitination and deubiquitination is essential for controlling the turnover of modified proteins. Thus, deubiquitinating enzymes that cleave ubiquitin from substrate proteins are equally important for determining the ubiquitination level of group I receptors, although studies of this kind are limited. One study found that a deubiquitinating enzyme, Usp4, bound to the CT region of A_2A_ adenosine receptors and deubiquitinated the receptors [20]. However, the Usp4 interaction was not seen with the mGlu5 receptors. Future studies are warranted to identify deubiquitinating enzymes that act to deubiquitinate group I receptors as well as other subgroups of mGlu receptors.

## Group III mGlu Receptors

Group III receptors include four subtypes (mGlu4/6/7/8). Emerging evidence reveals that presynaptic mGlu7 receptors are subject to ubiquitination [21,22]. Dimers and multimers although not monomers of recombinant mGlu7 receptors were ubiquitinated at multiple lysine sites on both CT and an intracellular loop in transfected HEK293T cells [21] (Table 1). Ubiquitination of mGlu7 was constitutively active and could be upregulated in an activity-dependent fashion in response to agonist stimulation. Native mGlu7 receptors were also sensitive in its ubiquitination to ligand stimulation in cultured rat cortical neurons. In searching for an E3 ligase specific for mGlu7 ubiquitination, Nedd4 (neural precursor cell-expressed developmentally down-regulated 4) was found to play such a role in constitutive and agonist-stimulated ubiquitination. Moreover, β-arrestins participate in the ligand-stimulated ubiquitination of mGlu7 by recruiting Nedd4 to the activated receptors.

Ubiquitin contains seven lysine residues (K6, K11, K27, K29, K33, K48, and K63) and an N-terminal methionine (M1) that serve as points for forming polyubiquitination chains. K48- and K63-linked ubiquitin chains are the two best-characterized types of ubiquitin chains. The former targets the substrate protein to the proteasome for degradation, whereas the latter is not associated with the proteasome and instead may direct the substrate protein to other processes, including endocytic trafficking. The K63-linked ubiquitin chain was observed in mGlu7 receptors [21]. Thus, mGlu7 ubiquitination is likely involved in receptor endocytosis. The finding that surface-expressed but not intracellular mGlu7 receptors underwent the agonist-induced ubiquitination [21] seems to imply that surface mGlu7 receptors are sorted to go through the endocytic route. In support of this, an E1 activating enzyme inhibitor and siRNA knockdown of Nedd4 reduced the agonist-induced mGlu7 ubiquitination and endocytosis. In addition to the K63-linked chain, the K48-linked ubiquitin chain was present in mGlu7 receptors, indicating a role of ubiquitination in signaling mGlu7 to degradation. Indeed, agonist stimulation induced a Nedd4/ubiquitination-sensitive decrease in total mGlu7 protein levels [21]. The proteasome inhibitor MG132 reduced this decrease, suggesting a role of the proteasome in the degradative event. In addition, the lysosome inhibitor leupeptin produced the similar effect as MG132. Thus, ubiquitination also sorts mGlu7 into early and late endosomes, followed by degradation in lysosomes.

## Other Synaptic Proteins Associated with mGlu Receptors

In addition to mGlu receptor themselves, mGlu-associated proteins undergo ubiquitination. Ubiquitination of these proteins regulates their abundance and function, which in turn exerts a significant impact on the expression, subcellular distribution, and signaling of mGlu receptors. For example, Homer proteins are a family of adaptor proteins enriched at postsynaptic sites and are associated with multiple synaptic proteins, especially the group I receptors. A short-form Homer protein, i.e., Homer1a, was robustly ubiquitinated in HEK293T cells [23]. This ubiquitination targeted Homer1a to degradation by the proteasomal but not lysosomal pathway under normal conditions. Inhibition of proteasomes thus enhanced Homer1a protein levels and furthermore increased delivery of Homer1a to synaptic sites in cultured hippocampal neurons [24]. In contrast to Homer1a, long-form Homer proteins (Homer1b/c, Homer2, and Homer3) were resistant to proteasomal degradation. Homer was discovered as a postsynaptic protein that specifically binds to a consensus proline-rich sequence in the CT region of group I mGlu receptors [25]. Activity-induced Homer1a can disrupt the cross-linking action of constitutively expressed long Homer isoforms to modulate surface expression of group I receptors and group I receptor-mediated Ca^2+^ signaling and other activities [26,27]. It is likely that activity-dependent ubiquitination and degradation of Homer1a serve as a molecular mechanism underlying the regulation of expression and function of Homer1a in relation to the modulation of glutamatergic transmission and synaptic plasticity.

The activity-regulated cytoskeleton-associated protein Arc/Arg3.1 (Arc) is another synaptic protein that is associated with group I receptor-mediated synaptic plasticity and is regulated by ubiquitination. As an immediate early gene product, Arc is rapidly induced via an increase in its transcription and translation in response to changing cellular and synaptic input. Induced Arc then regulates various activities at glutamatergic synapses. For instance, in response to DHPG, Arc is induced to facilitate endocytosis of AMPA receptors, an event implicated in group I mGlu-LTD [28]. Many newly synthesized synaptic proteins are subject to ubiquitin-dependent turnover to ensure a tight control of their quantities at synaptic sites and a discrete temporal window for their actions [29,30]. Arc is among these highly dynamic proteins and was ubiquitinated after synthesis at K268 and K269 sites in heterologous cells or neurons [31–33] (Table 1). Ubiquitination of Arc promoted Arc to proteasomal degradation. Given that Arc-mediated endocytosis of AMPA receptors is a core element in group I mGlu-LTD, Arc ubiquitination may negatively regulate the mGlu-LTD. In fact, enhanced Arc ubiquitination by overexpression of an E3 ubiquitin ligase (Triad3A) reduced DHPG-induced synaptic depression in cultured hippocampal neurons, whereas knockdown of Triad3A produced the opposite effect [33]. In a mutant mouse line in which ubiquitin-dependent degradation of Arc is disabled, DHPG induced a higher level of Arc expression and a higher amplitude of LTD at the Schaffer collateral–commissural pathway in the CA1 region of hippocampal slices [34]. Similarly, disrupting Arc ubiquitination enhanced the DHPG-stimulated Ca^2+^ release from the endoplasmic reticulum [35]. In *in vitro* and *in vivo* seizure models, the reduction of ubiquitin-dependent degradation of Arc enhanced the hippocampal group I mGlu-LTD *in vitro* and reduced it *in vivo* [36]. In addition to LTD, Arc ubiquitination supports the induction and expression of long-term potentiation (LTP) in the hippocampal CA1 area [37]. Of note, inconsistent results were reported regarding the role of Ube3A (also known as E6AP), an E3 ubiquitin enzyme, in binding and ubiquitinating Arc [28].

Unlike its effects in the CA1 area, DHPG alone did not induce LTD at the mossy fiber pathway in the CA3 region of rat hippocampal slices [38]. Co-activation of the G protein-coupled estrogen receptor 1 was required for the agonist to induce LTD. Notably, this form of mGlu-LTD is associated with the DHPG-induced rapid ubiquitination and proteasomal degradation of the AMPA receptor GluA1 subunit but not Arc. In addition to GluA1, mGlu5 regulates glycine receptor ubiquitination in an activity-dependent manner. In mouse spinal cord dorsal horn neurons, activation of postsynaptic mGlu5 receptors by DHPG in the presence of mGlu1 antagonist or by the mGlu5 agonist CHPG stimulated ERK to phosphorylate the glycine receptor α1^ins^ subunit at an S380 site. This phosphorylation facilitated monoubiquitination of α1^ins^ subunits, leading to α1^ins^ endocytosis and inhibition of overall glycinergic transmission [39].

The protein interacting with C-kinase 1 (PICK1) is a synaptic scaffold protein that interacts with a number of synaptic receptors, transporters, and ion channels [40]. Among these PICK1-interacting proteins is the presynaptic mGlu7 receptor. As a PDZ-domain-containing protein, PICK1 directly binds to the PDZ ligand at the extreme CT of mGlu7 [41–46]. Such binding is critical for the regulation of phosphorylation, presynaptic clustering, synaptic localization, surface stability, and signaling of mGlu7 receptors. Notably, PICK1 is among synaptic scaffold proteins subjected to ubiquitination. Parkin, an E3 ubiquitin ligase, bound to PICK1 in a PDZ-dependent manner, which enables Parkin to monoubiquitinate but not polyubiquitinate the target [47]. Consistent with the notion that monoubiquitination could regulate the function of tagged proteins without necessarily sorting them to degradation by the proteasome, Parkin reduced the PICK1-dependent potentiation of acid-sensing ion channels, while it did not promote PICK1 to proteasomal degradation. Given the robust linkage of PICK1 with mGlu7, the Parkin-mediated monoubiquitination of PICK1 might have a profound influence over mGlu7 receptors, an interesting topic to be investigated in future studies. In addition to PICK1, Rab3-interacting molecule 1 (RIM1) is a scaffold protein in the active zone. One study found that RIM1 was ubiquitinated by a presynaptically-localized E3 ubiquitin ligase, SCRAPPER, and was degraded in the proteasome [48]. Further studies observed that SCRAPPER plays critical roles in regulating the thresholds of LTP/LTD in mouse hippocampal CA3-CA1 synapses [49] and in determining the formation of hippocampus-dependent fear memory in mice [50]. Notably, RIM1, like PICK1, is a known PDZ-domain-containing protein. Whether RIM1 binds to the PDZ ligand of any presynaptic mGlu receptors is currently unclear.

## Conclusions

Presynaptic mGlu7 receptors undergo constitutive and agonist-induced ubiquitination. Postsynaptic group I receptors are also ubiquitinated. Agonist stimulation triggers polyubiquitination of mGlu1 to promote a negative feedback regulation. Like mGlu receptors, a set of mGlu-associated synaptic proteins, including Homer1a, Arc and PICK1, are subject to ubiquitination. Specific E3 ubiquitin ligases catalyze the ubiquitination of a specific mGlu substrate. All types of ubiquitination (mono- versus polyubiquitination) are physiologically relevant. They could either direct mGlu receptors/synaptic proteins to degradation via the UPS or autophagy/lysosomal pathway or play nondegradative roles in the regulation of their trafficking, distribution patterns, protein-protein interactions, and functions.

It should be pointed out that preclinical studies on mGlu ubiquitination biology are still at an early stage. Several lines of future studies are needed to gain in-depth understanding of biochemical and physiological properties of mGlu ubiquitination in a constitutive state or in response to changing synaptic input. First, additional E3 ubiquitin ligases may be discovered to possess the ability to ubiquitinate mGlu receptors. The nature that E3 ligases confer specificity of substrate proteins is noteworthy. Additionally, deubiquitination enzymes are worth more attention as little is known about their roles in the delicate balance controlling mGlu ubiquitination. Second, mGlu receptors are knowingly subject to other types of subtle posttranslational modifications such as phosphorylation, sumoylation, etc., in addition to ubiquitination [51,52]. Active crosstalk is believed to occur among these different modification processes. Future studies can aim to explore and characterize their potential crosstalk activities. Finally, timely attempts are much needed to link the knowledge of mGlu ubiquitination to brain illnesses. Ubiquitination of mGlu receptors and associated proteins may be a vulnerable event and may undergo adaptive changes during the development of chronic brain diseases, which contributes to the receptor and synaptic plasticity critical for the pathogenesis and symptomology of various brain disorders.

## Figures and Tables

**Table 1.** Ubiquitination of mGlu receptors and associated synaptic proteins.

Protein	Ubiquitination site	E3 ligase	Physiological impact
mGlu1	Multiple lysine sites in intracellular loops and CT, including K1112 in CT	Siah1A	Increase mGlu1 degradation via proteasomes and promote agonist-induced endocytosis
mGlu5	Multiple lysine sites in intracellular loops and CT	Siah1A	Accelerate mGlu5 degradation via lysosomes and proteasomes
mGlu7	K688 and K689 in second intracellular loop and multiple lysine sites in CT	Nedd4	Increase mGlu7 degradation via proteasomes and lysosomes and promote agonist-induced endocytosis
Homer1a	ND	ND	Increase Homer1a degradation via proteasomes
Arc	K268 and K269	Triad3A	Promote Arc to proteasomal degradation, negatively regulate mGlu-LTD, and reduce group I agonist-stimulated Ca^2+^ release
PICK1	Monoubiquitination rather than polyubiquitination	Parkin	Reduce PICK1 function in a proteasome-independent manner
RIM1	ND	SCRAPPER	Increase proteasomal degradation of RIM1

ND: Not Determined. See text for other abbreviations.
